# The roles of veterinarians in meeting the challenges of health and welfare of livestock and global food security

**Published:** 2012

**Authors:** Sigfrido Burgos Cáceres

**Affiliations:** *Current Address: 28957 Sampson Avenue, Orange Beach, Alabama, 36561-4055, USA*


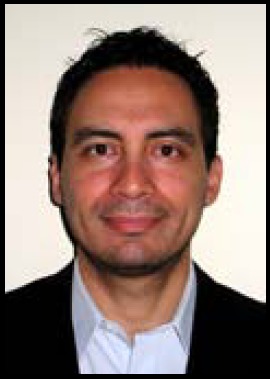


The classical view of a veterinarian as a person qualified to practice veterinary medicine has lead most people to think that their clinical practices are related to the prevention, cure or alleviation of pain and treatment of injuries in animals, especially domestic animals. Though these roles are very important, the contemporary roles undertaken by veterinarians go far beyond these more visible tasks, and this is the reason why there is a need for far greater awareness in the public eye. It must be recognized that as the world becomes intricately inter-connected and more complex, so are the various obligations and responsibilities that veterinary professionals must undertake. 

Over the years veterinary professionals have played significant and contributory roles in animal and human health and welfare, biomedical research, food quality, food safety, food security, ecology, ethology, epidemiology, microbiology, parasitology, pathology, physiology, psychology, radiology, research and development of pharmaceuticals, remedies, vaccines, and toxicology; also as educators, trainers, and policymakers, and also interlinked with wildlife conservation efforts and the protection of the environment and biodiversity. As challenges have risen, veterinarians have found ways to adapt given that their knowledge and training makes them multifunctional professionals. This aids societies so that its animals stay healthy and productive. It is not surprising that becoming a veterinarian is a highly popular career choice.

Recognition that healthy and productive livestock make important contributions to food production, income generation, job creation, economic growth, and poverty alleviation is often overlooked or taken for granted. Yet, on average, livestock contribute some 40 percent of agricultural GDP.^1^

As the world population grows and middleclass incomes rise, demand for livestock products increases—a consumption boom shaped by two decade of rapid economic growth and globalization. But there are certain high impact diseases that do not allow animal husbandry to flourish. Less dramatic diseases also impact the performance of farm animals, leading to lower production efficiencies and associated financial losses due to mortality and morbidity. Also, poor animal health in turn negatively influences animal welfare. In fact, studies have demonstrated that there is a direct correlation between the quality of livestock production and the provision of veterinary services.^2^ Given that food animals support the livelihoods and nourishment of almost a billion people, efforts should be directed at upholding food security.

 In this regard, farm-oriented veterinary professionals should advise farmers and owners of livestock or managers of animal production systems on the most appropriate herd health management practices according to local and regional agro-ecological contexts. This extends beyond the treatment of animal illnesses or the implementation of preventive measures, such as strengthened biosecurity, programmed de-worming or vaccination, but also addresses housing, nutrition, cleaning, and environmental sanitation. The correct undertaking of latter practices will likely reduce the usage of veterinary medications and care, thereby reducing input and labor costs, which in turn positively influences farm productivity and profit margins.

The veterinarian’s job extends to advisory and hands-on tasks throughout the value chain such as, for instance, in ante- and post-mortem inspections and further food safety interventions to ensure a safe and wholesome food supply to consumers. Also, veterinarians make sure that healthy animals are exported, imported, and distributed, thus preventing the risk of introducing detrimental, high-impact diseases into distant regions or neighboring countries.

Experienced veterinary professionals are also involved in developing guidelines, norms, and standards. For example, the Livestock Emergency Guidelines and Standards (LEGS) have been developed as a set of international guidelines and standards for the design, implementation, and assessment of livestock interventions to assist people affected by humanitarian crises,^3^ while other veterinarians are involved in overlapping tasks related to animal disease emergencies, disaster relief, risk management, or threat minimization.^4^ Other veterinarians are involved in setting up and publishing health standards for international trade in animals and animal products, disease diagnosis and vaccines (e.g. OIE Animal Health Codes and Manuals).^5^^,^^6^

As for global food security, FAO and WFP^7^ estimates indicate that a total of 925 million people were undernourished in 2010, compared with 1.023 billion in 2009. Most of the decrease was in Asia, with 80 million fewer hungry, but progress was also made in sub-Saharan Africa, where 12 million fewer people went hungry. However, the number of hungry people is higher in 2010 than before the food and economic crises of 2008-2009. Improving food security requires going beyond short-term responses in order to protect and promote people’s livelihoods over the longer term, with agriculture and the livestock subsector playing pivotal roles.

Healthy and productive livestock produce a wide variety of food products for direct and indirect human consumption and processing. These products include blood, eggs, meat and meat products, milk and dairy products, viscera as well as rendering by-products such as brains, ears, feet, skin, testicles, tongues, and udders. Bones, horns, and leathers are other by-products that feed into value-adding systems for commercial purposes. The food and income from healthy animals empower farmers of all scales to embrace the advantages, education, openings, opportunities, leverages, and strengths they need to produce more food and income to feed an ever-increasing world population. Also, animal food products from healthy and productive livestock improve farmers’ access to both domestic and international markets.^8^ It is for this very reason that the provision of veterinary services by national and regional agencies is a key component of efforts supporting sustainable production of food from animal origins and to the success of farm operations. 

Though livestock products provide quality and nutritious foods for urban, peri-urban, and most rural consumers, in certain areas of the world there are cultural and socioeconomic determinants behind the consumption of bush-meat. For instance, for hundreds of years, African societies have been eating wildlife for nourishment. Studies show that between 30 to 80 percent of protein intake in rural households in Central Africa comes from wild meat. More specifically, it is estimated that over 4.5 million tons of bush-meat are extracted annually from the Congo basin. Also, the financial returns from hunting wildlife are higher than average local wages given that in some parts of Africa bush-meat prices can be up to 10 times higher than beef, thus providing strong incentives to engage in this activity regardless of risks.^9^ In view of this, wildlife veterinarians, biologists, and socio-economists can make important contributions to the understanding of these needs, as well as to better address animal (and human) health issues.^10^

Other contributions to food security can be shown through the multidimensional benefits of eradicating Rinderpest from the face of the earth: an achievement of international organizations and partners under the Global Rinderpest Eradication Programme celebrated on 28 June 2011.^11^ Rinderpest eradication in Africa represents over USD1 billion in savings to local economies invested in livestock. Also, rural veterinarians play preponderant roles in the fight against H5N1 highly pathogenic avian influenza since it surfaced in Southeast Asia in late 2003, affecting millions of people’s health, livelihoods, and nutrition.

Nowadays, a vast number of veterinarians working for intergovernmental and nongovernmental organizations are also active in addressing African swine fever, rabies, brucellosis, contagious bovine pleuropneumonia, foot-and-mouth disease, haemorrhagic septicaemia, peste des petits ruminants, Rift Valley fever, and African trypanosomosis, as well as improving information systems, field investigation methods, laboratory networks and quality controls, risk and threat analysis and mitigation, provisions of vaccines and treatments, and rapid responses to emergencies and humanitarian crises.^12^

When zoonotic diseases strike any given geographical location, veterinary professionals are the first source of informed opinion on veterinary issues for governments, veterinary surgeons, the media, civil society organizations and charities, action and consumer groups, and the public. It has long been recognized that early response to animal diseases with the potential to result in epidemic events is of tremendous benefit for generations to come. To this end, veterinary scientists, scholars, and professors are the leading providers of veterinary news, research, and information, including clinical and scientific developments to the wider veterinary community and other interested disciplines in science. Closely aligned with information dissemination, the improvement of the welfare of all animals through veterinary science, education, and debate among interested parties has resulted in highlighting and promoting discussions on pertinent welfare issues which are taken forward by other institutions and society. 

Veterinary professionals also share responsibilities for biosecurity. This is a joint effort that must be shared by all who have obligations for animals and/or animal products, both farm and companion animals. The conventional barriers that nature provides can no longer be relied upon to exclude dissemination from country to country. In this view, the veterinarian is a sentinel for the early detection of, and early response to, accidental or deliberate introduction of exotic diseases. The veterinarian is, in fact, a key line of defense that society counts on against agro-terrorism and bioterrorism.^13^ In sum, veterinary professionals are key players on biodefense, and thus for national security, food chain safety, and animal and human welfare.
